# SIRT1 activation and its effect on intercalated disc proteins as a way to reduce doxorubicin cardiotoxicity

**DOI:** 10.3389/fphar.2022.1035387

**Published:** 2022-11-04

**Authors:** Ekaterina Podyacheva, Yana Toropova

**Affiliations:** Almazov National Medical Research Centre, Ministry of Health of the Russian Federation, Saint Petersburg, Russia

**Keywords:** adherens junctions, desmosomes, doxorubicin cardiomyopathy, gap junction, heart failure, intercalated discs, NAD+, sirtuins

## Abstract

According to the World Health Organization, the neoplasm is one of the main reasons for morbidity and mortality worldwide. At the same time, application of cytostatic drugs like an independent type of cancer treatment and in combination with surgical methods, is often associated with the development of cardiovascular complications both in the early and in the delayed period of treatment. Doxorubicin (DOX) is the most commonly used cytotoxic anthracycline antibiotic. DOX can cause both acute and delayed side effects. The problem is still not solved, as evidenced by the continued activity of researchers in terms of developing approaches for the prevention and treatment of cardiovascular complications. It is known, the heart muscle consists of cardiomyocytes connected by intercalated discs (ID), which ensure the structural, electrical, metabolic unity of the heart. Various defects in the ID proteins can lead to the development of cardiovascular diseases of various etiologies, including DOX-induced cardiomyopathy. The search for ways to influence the functioning of ID proteins of the cardiac muscle can become the basis for the creation of new therapeutic approaches to the treatment and prevention of cardiac pathologies. SIRT1 may be an interesting cardioprotective variant due to its wide functional significance. SIRT1 activation triggers nuclear transcription programs that increase the efficiency of cellular, mitochondrial metabolism, increases resistance to oxidative stress, and promotes cell survival. It can be assumed that SIRT1 can not only provide a protective effect at the cardiomyocytes level, leading to an improvement in mitochondrial and metabolic functions, reducing the effects of oxidative stress and inflammatory processes, but also have a protective effect on the functioning of IDs structures of the cardiac muscle.

## Introduction

The neoplasm is one of the main reasons for morbidity and mortality worldwide (de Martel et al., 2020; Markham et al., 2020; Wild et al., 2020). Аpplication of cytostatic drugs like an independent type of cancer treatment and in combination with surgical methods, is often associated with the development of complications both in the early and in the delayed period of treatment (Bray et al., 2018). Anthracycline-induced cardiomyopathy was identified by oncologists and cardiologists in the early 90s as a serious cardiovascular complication of chemotherapy. Doxorubicin (DOX) is the most commonly used cytotoxic anthracycline antibiotic. DOX has been known since the late 1960s and is now widely used in oncological practice to treatment of a wide range of cancer types, such as breast cancer, thyroid cancer, and bladder cancer, gastric cancer, ovarian cancer, non-Hodgkin’s lymphomas, trophoblastic tumors and others (Vejpongsa and Yeh 2014a; Kleinerman, 2014). However, dose-dependent anthracycline-related cardiotoxicity, leading to the development of progressive heart failure and irreversible cardiac dysfunction, severely limits DOX use. Сumulative doses of DOX as 400, 550 and 700 mg/m2 using in clinical practice cause a heart failure in 5%, 28% and 48% cases, respectively (Swain et al., 2003; Vejpongsa and Yeh 2014b; Vejpongsa and Yeh 2014a; Carvalho et al., 2014). Symptomatic therapy is used for prevention of anthracyclines cardiotoxic side effects, but it provides just a short-term effect. Dexrazoxane, an ethylenediaminetetraacetic acid analogue, is the only drug approved for clinical use to reduce DOX-induced cardiotoxicity. Dexrazoxane disrupts the formation of the anthracycline-Fe complex and thereby prevents the appearance of Fe-containing free radicals (Minotti et al., 2004). In this regard, the development of approaches focused on the prevention and treatment of doxorubicin cardiomyopathy remains an urgent problem in modern medicine.

Cardiac muscle is a network of cardiomyocytes connected by intercalated discs (IDs), which are ensure the structural, electrical, metabolic unity of the heart is ensured, allowing the heart muscle to act in a coordinated manner during its contraction and excitation. The myocardial IDs are one of the key structures involved in the cardiovascular diseases development of various aetiology, including DOX-induced cardiomyopathy, due to their integral role in the implementation of intercellular communication, as well as in ensuring the mechanical and electrical activity of the heart (Zhao et al., 2019). IDs can act as a “sensor of inadequate functional activity” at the level of cardiomyocytes. IDs additionally stimulate the growth of myofibrils in cells when the transmission of nerve impulses, and therefore the coordinated contraction of cardiomyocytes is impaired due to defects, mutations or changes in the expression levels of various cytoskeletal proteins (Perriard et al., 2003).

SIRT1 are part of the Silent Information Regulator 2 (Sir2) family of proteins and are nicotinamide adenosine dinucleotide (NAD+)-dependent deacetylase. One is able to regulate various cellular functions, energy metabolism and resistance to oxidative stress, inflammatory processes, mediating cell survival. SIRTs are one of the key enzymatic systems that respond to DNA damage caused by excessive accumulation of free radicals (Imai and Guarente 2014). Poly-ADP-ribose polymerases (PARPs) compete with them for binding site of damaged DNA. PARPs, localized in the nucleus, respond to DNA breaks and promote the process of its repair. They are able to synthesize the polymer poly (ADP-ribose). ADP-ribose acts as a signal and at the same time as a scaffold with which the proteins involved in the repair interact (Bai and Cantó 2012). The donor of ADP-ribose is NAD+. An increase in mitochondrial activity leads to an increase in the concentration of both PARP and SIRT1 (Bai and Cantó, 2012; Módis et al., 2012). While prolonged PARPs activation leads to the depletion of NAD + cellular pools and to a decrease in mitochondrial function. Whereas SIRT activation and PARP-1/-2 inhibition (Alcendor et al., 2007; Módis et al., 2012) allows to save mitochondrial activity. In addition, SIRT1 mediate a large number of signaling pathways that are involved in such processes as: fibrosis development, hypertrophy (SIRT1/PGC-1α) (Dolinsky, 2017), apoptosis regulation (SIRT1/p53, SIRT1/p66Shc) (Boengler et al., 2019), autophagy (SIRT1/AMPK) (Gratia et al., 2012), pyropoptosis (SIRT1/NLRP3) (Bakker et al., 2014), endoplasmic reticulum stress (SIRT1/eEF2K/eEF2/eIF2α) (Pires Da Silva et al., 2020), angiogenesis (SIRT1/ FOXO1, SIRT1/ Notch) (Knight and Milner 2012; Cao et al., 2014; Chen et al., 2020). Therefore, the SIRT1 activation can be considered as one of the pathogenetically substantiated effects on the heart muscle damaged by DOX.

It is important to note that a number of environmental factors, such as bisphenol A, nanoparticles, endosulfan, can reduce the expression of Sirt1 in human cells, increasing the risk of cardiovascular disease. For example, bisphenol A is the most well-known endocrine disruptor, co-incubated with DOX can exacerbate cardiotoxicity in cardiomyoblasts by increasing the release of pro-inflammatory interleukins and enhancing lipid peroxidation (Quagliariello et al., 2018).

Many studies explore the mechanisms of the SIRT1 protective effect on the heart muscle damaged by DOX (Chang and Guarente 2014; Dolinsky, 2017; Meng et al., 2017; D’Onofrio et al., 2018; Wallace et al., 2020). Drugs such as butyrate and resveratrol, aimed at activating sirtuins, are used in anticancer, cardioprotective therapy (Brookins Danz et al., 2009; Cappetta et al., 2016; Quagliariello et al., 2018; Zhang et al., 2019). Resveratrol contributes to a decrease in ROS production, due to an increase in MnSOD activity, promotes inhibition of the TGF-β/SMAD3 pathway, inhibition of p38MAPK phosphorylation and caspase-3 activation by activating SIRT1 (Becatti et al., 2012; Ruan et al., 2015). Thus, resveratrol prevents the cardiac fibrosis development, the death of cardiomyocytes in DOX-induced cardiomyopathy model. Butyrate can inhibit proliferation and induce apoptosis in HCT116 cells by deactivating mTOR/S6K1 signaling, possibly through inhibition of SIRT1 (Cao et al., 2014). Butyrate also induces ROS-mediated apoptosis by modulating the miR-22/SIRT-1 pathway in liver cancer cells (Pant et al., 2019). It can be assumed that SIRT1 can positively manifest itself in solving the problem of the development of DOX-induced cardiotoxicity. SIRT1 can not only provide a protective effect at the cardiomyocytes level, leading to an improvement in mitochondrial and metabolic functions, reducing the effects of oxidative stress and inflammatory processes, but also have a protective effect on the functioning of IDs structures of the cardiac muscle. Meanwhile, the last mechanism of the possible cardioprotective effect of SIRT1 is practically not covered in the literature.

This review is aimed at structuring information on the molecular basis of the involvement of IDs proteins in pathological changes in the heart muscle under DOX exposure, as well as forming the basis for the hypothesis of the preventive and therapeutic effect of SIRT1 activation on the development of DOX-induced dilated cardiomyopathy and chronic heart failure through the influence on IDs proteins.

## Article search and selection strategy

The search for published articles and reviews in peer-reviewed open access journals was carried out using the following databases: PubMed, Google Scholar. In addition, we used existing abstracts of articles or entire articles on ResearchGate without open access. Most of the peer-reviewed articles have been published within the past 10–15 years. Older work was seen rather as a source of fundamental discoveries. Additional databases were also searched through Google using the following keywords: intercalated discs, dilated cardiomyopathy, gap junction, desmosomes, adherens junctions, doxorubicin, heart disease, NAD+, Sirtuins, signaling and similar.

## Modern view on the mechanisms of development of doxorubicin-induced cardiomyopathy

**FIGURE 1:**
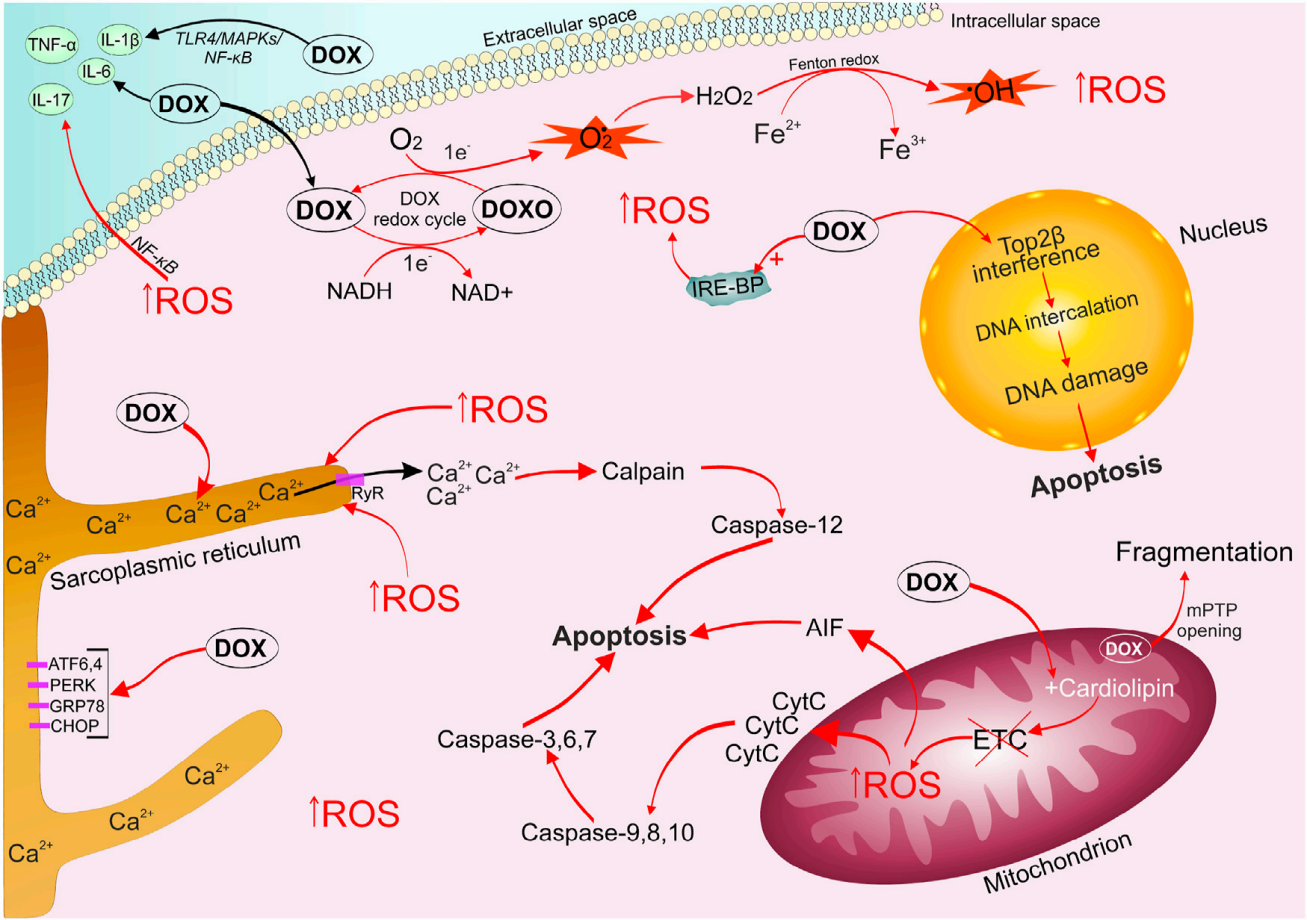
Mechanisms of doxorubicin-induced cardiomyopathy development. DOX is easily captured by cells, it undergoes one-electron reduction with the formation of a superoxide radical. The alternation of quinone and semiquinone DOX structures triggers the generation of a large amount of ROS. DOX leads to disruption of the electron transport chain, which triggers mitochondrial signaling pathways of apoptosis. The emerging oxidative stress causes an imbalance of calcium ions in the sarcoplasmic reticulum, the opening of mPTP, which ultimately activates the work of apototic caspases. DOX stimulates the accumulation of unutilized free iron in the cell, by binding to intracellular iron sequestration proteins, which enhances the ROS formation cycle. DOX and oxidative stress contribute increased production of inflammatory factors ((IL-1β), IL-6, IL-17, TNF-α) *via* activation of (TLR4)/MAPKs/NF-κB pathways. DOX, captured by the cell nucleus, binds to Top2β, forming a triple DOX-Top2β-DNA complex, which inhibits DNA replication, stops the cell cycle in G1 / G2 and initiates programmed cell death. ATF6/4, activating transcription factor 6/4; CHOP, C/EBP homologous protein; DOX, doxorubicin; DOXO, semiquinone form of doxorubicin; ETC, electron transport chain; GRP78, 78-kDa glucose-regulated protein precursor; IL-6/17/1β, interleukin 6/17/1β; IRE-BP, iron-responsive element-binding proteins; MAPKs, mitogen-activated protein kinases; mPTP, mitochondrial permeability transition pore; NF-κB, nuclear factor kappa-light-chain-enhancer of activated B cells; PERK, (PKR)-like endoplasmic reticulum kinase; ROS, reactive oxygen species; TLR4, toll-like receptor 4; TNF-α, tumor necrosis factor; Top2β, topoisomerase 2β.

The mechanisms and patterns of anthracycline-induced cardiotoxicity development are still being studied (Maral et al., 1967; Jaenke, 1974; Johansen, 1981; Herman et al., 1985; Herman and Ferrans 1997; Aykan et al., 2020; Chakouri et al., 2020; Sharma et al., 2020; Podyacheva et al., 2021). A generalized modern idea of them is presented in Figure 1.

Oxidative stress is characterized by an imbalance between the production of reactive oxygen species (ROS) and the ability of the antioxidant defense system to inactivate them (Renu et al., 2018; Corremans et al., 2019; Adhikari et al., 2021). Mitochondria are the first cellular components susceptible to DOX damaging effects. DOX is a tetracyclic aglycone linked with an amino sugar. The quinone fragment of one of the tetracyclic rings can accept electrons from donors presented in the cell (nicotinamide adenine dinucleotide phosphate-cytochrome P450 reductase, reduced nicotinamide adenine dinucleotide dehydrogenase, xanthine oxidase, cytochrome b5 reductase). Therefore, the quinone structure of DOX passes into the formation of an intermediate semiquinone free radical due to one-electron reduction (Minotti et al., 2004; Vejpongsa and Yeh 2014b). Further, the unpaired electron of semiquinone can be transferred to an oxygen molecule to form a superoxide radical (O2•), while the tetracyclic ring returns to the original quinone structure. O2• is a very active electron donor, which in the cell undergoes dismutation through the enzymatic activity of superoxide dismutase, turning into a less toxic molecule of hydrogen peroxide (H2O2) and O2. In turn, H2O2 and O2• potentiate the formation of highly toxic hydroxyl radical (OH•) (Haber-Weiss reaction) (Renu et al., 2018). Thus, the alternation of quinone and semiquinone DOX structures can produce a large amount of ROS from a relatively small amount of the drug. Lin et al. wrote that NAD-phosphate (NADPH) oxidase 2 (NOX2) and NOX4 may also be involved in DOX stimulation of increased ROS production and subsequent oxidative stress in cardiomyocytes (Lin et al., 2019). Mitochondria are most intensively exposed to ROS-mediated effects of DOX, as they are the main working structure of cardiomyocytes, which produces a huge amount of energy for the normal functioning of the heart muscle (Octavia et al., 2012). It should be noted that cardiomyocytes are characterized by a relatively weak antioxidant defense system compared to other body cells. ROS react with DNA, proteins, and lipids, which ultimately leads to lipid peroxidation, DNA damage, disruption of heart-specific gene expression programs, and necrotic/apoptotic death (Deng and Wojnowski 2007; Octavia et al., 2012).

It is worth noting that DOX has a high irreversible affinity for cardiolipin. Cardiolipin is a protein localized on the inner membrane of the mitochondria and contributing to the normal functioning of electron transport chain proteins (Kashfi et al., 1990; Schlame et al., 2000; Songbo et al., 2019). This property of DOX leads to disruption of the electron transport chain Complex I, which contributes to an increase in the generation of ROS (Wallace et al., 2020). Oxidative stress has a damaging effect on the mitochondria, causing the opening of the mitochondrial permeability transition pore (mPTP). The opening of mPTP allows free passage of solutes up to 1.5 kDa into the compartment, which can lead to the collapse of the inner membrane potential, stopping the synthesis of mitochondrial adenosine triphosphate (ATP) and disconnection of the respiratory chain, promoting swelling and fragmentation of mitochondria (Kwong and Molkentin, 2015).

DOX is known to increase the production of inflammatory factors such as interleukin-1β (IL-1β), IL-6, IL-17 (Sauter et al., 2011), p38 mitogen-activated protein kinase (p38 MAPK)/NF-κB (Guo et al., 2013), and tumor necrosis factor alpha (TNF- α) in the heart muscle, leading to the development of an inflammatory reaction. Moreover, oxidative stress directly induces an inflammatory response through the nuclear factor kappa B (NF-κB) activation (Quagliariello et al., 2018). Zhang et al. showed that DOX can promote excessive activation of the Toll-like receptor (TLR4)/MAPKs/NF-κB pathway (Zhang et al., 2019), carrying out inflammation.

DOX activates cell death through activation of necroptosis (Choi et al., 2009). TNF-α activates the protein TNFR-associated death protein (TRADD) *via* TRFR1 and phosphorylates receptor-interacting serine/threonine-protein kinase 1 (RIPK1), which in turn phosphorylates RIPK3 to form the necroptosomes. Necroptosome phosphorylates the mixed lineage kinase domain-like protein (MLK1), ruptures the plasma membrane and releases inflammatory factors, triggering an immune response and leading to cell death (Yu et al., 2020; Christidi and Brunham, 2021). mPTP opening process, caused by the toxic action of DOX, also leads to necroptosis and apoptosis.

Pyroptosis is characterized by increased inflammation and activation of caspase-1, caspase-3, caspase-4 and caspase-11, as well as the NLR family pyrin domain containing 3 (NLRP3). Pyroptosis has been playing an important role in the pathogenesis of DOX-induced cardiomyopathy since 2001 (Ma et al., 2020). Stimulation of caspase-1,3,4,11 and NLRP3 leads to cleavage of gasdermin D (GSDMD)/GSDME and rupture of the plasma membrane, which causes the release of interleukin-1 beta (IL-1β) and IL-18. Moreover, DOX triggers GSDME-dependent pyroptosis through increased BCL2/adenovirus E1B 19 kDa protein-interacting protein 3 (Bnip3) expression in mitochondria, which activates caspase 3 (Zheng et al., 2020). Several studies have shown that sirtuin 1 stimulation (eg, resveratrol) inhibits NLRP3 and protects cardiomyocytes from DOX-induced pyroptosis (Ding et al., 2020; Han et al., 2020; Sun et al., 2020).

Anthracyclines, as well as the oxidative stress, can cause an imbalance in Ca2+ homeostasis in the sarcoplasmic reticulum of cardiomycytes. This event leads to disruption of the coordinated interaction between excitation and contraction (Tabas and Ron 2011). Calpains are activated in response to Ca2+ leakage. Calpains are proteases that respond to the dysregulation of calcium ions and are able to destroy titin (the main component of the cardiac sarcomere), which supports myocardial contractility (Octavia et al., 2012). Caspase-12 cleavage is triggered in response to the calpains activation. Caspase-12 initiates the mitochondrial pathway of apoptosis. DOX can directly increase the expression of endoplasmic reticulum (ER) stress-related proteins such as protein RNA-like ER kinase (PERK), activating transcription factor 6 (ATF6), ATF4, a 78-kDa glucose-regulated protein precursor (GRP78), and homologous protein C/EBP (CHOP), causing ER stress leading to apoptosis (Fu et al., 2016).

A number of studies have demonstrated the ability of DOX to interact with proteins that sequester and bind intracellular iron, leading to the formation of the doxorubicinol metabolite complexes with the Fe–S group of cytoplasmic aconitase/IRP-1 (iron regulatory protein). Thus, these complexes increase the stability of transferrin mRNA and preventing translation of iron sequestration proteins (May et al., 1980; Minotti et al., 1998). The accumulation of unutilized free iron enhances the cycle of ROS formation. In addition, ferroptosis also characteristic of DOX-induced cell death, which was first described by Dixon et al. (Dixon et al., 2012; He et al., 2021). Fang et al. report that ferroptosis mediates the pathogenesis of DOX-induced cardiotoxicity *via* the Nrf2/Hmox1 axis (Fang et al., 2019). Activation of Hmox1 by stimulating nuclear factor erythroid 2-related factor 2 (Nrf2) causes heme degradation in the heart and releases free iron. On the other hand, DOX can also inhibit the level of Glutathione peroxidase 4 (GPX4) in mitochondria, enhancing lipid peroxidation at the mitochondrial membrane (Li et al., 2021). Dexrazoxane is the only therapeutic drug approved by the Food and Drug Administration for clinical use to reduce anthracycline-related cardiotoxicity. Dexrazoxane action is based on the absorption of free radicals, excessively generated by the DOX-iron complex (Minotti et al., 2004).

DOX-induced cardiotoxicity is associated with the p53-dependent and intrinsic pathway of apoptosis (Papadopoulou et al., 1999). Phosphorylation of p53 leads to apoptosis of cardiomyocytes due to suppression of anti-apoptotic B-cell lymphoma 2 (Bcl-2), enhancement of pro-apoptotic Bcl-2-associated X-protein (Bax), release of Cyt C, apoptosis-inducing factor (AIF), activation of initiator and effector caspases, respectively (caspase-9, caspase-3) (Vedam et al., 2010). In general, such processes developing against the background of DOX exposure as oxidative stress, endoplasmic reticulum stress, mitochondrial dysfunction, a progressive inflammatory response, as well as an imbalance of calcium and iron ions in the cell, carry out the triggering of apoptosis.

The death of cardiac muscle cells initiates the development of diffuse interstitial fibrosis. Moreover, DOX alone can increase the expression of MMP1, TGF-β, and collagen in cardiac fibroblasts through activation of the phosphoinositide 3-kinase (PI3K)/Akt signaling pathway (Narikawa et al., 2019). It has been shown that DOX-induced activation of MMP2 causes remodeling of both the extracellular and intracellular matrix *via* proteolysis of cardiac titin and further lysis of myofilaments (Chan et al., 2016). It is been reported that angiotensin-II *via* AT-1R can cause fibrosis and inflammation (Sobczuk et al., 2020). It has been shown that DOX is able to cause an increase in the level of both angiotensin-II and angiotensin-converting enzyme (ACE) by 2–3 times (Okumura et al., 2002; Rashikh et al., 2014). Cardiac fibroblasts, responsible for the production of matrix protein, have AT-1R on their surface. Mitogen-activated protein kinases (MAPKs) and extracellular signaling kinases (ERK1/2) are activated after AT-1R stimulation by angiotensin-II, which leads to overexpression of collagen types 1 and 3 genes, as well as an increase in fibroblast density and their proliferation (Toko et al., 2002). Additionally, inflammation associated with elevated angiotensin-II levels at the cellular level can increase oxidative stress in cardiomyocytes and also can promote myocardial remodeling, carrying out hemodynamic dysregulation and as a result heart failure.

Autophagy makes a separate contribution to the development of DOX-induced cardiomyopathy. Aspects and mechanisms of this process require more detailed study, as the data vary. Some researchers discuss the ability of DOX to stimulate autophagy (Xu et al., 2018), while others talk about its suppression (Kawaguchi et al., 2012). The described contradictions may be associated with the use of different doses of the drug and different animal models, as well as different experimental conditions. It has been shown that the flow of autophagy may decrease with an increase of the DOX concentration in the cell, and as a result the cell’s self-purify ability is lost. These changes bring the cell closer to apoptosis (Pan et al., 2019).

In the cell nucleus, DOX has a destructive effect through irreversible binding to topoisomerase 2β (Top2β), which is expressed in resting cells such as adult cardiomyocytes. DOX blocks Top2β ability to ligate DNA double-strand breaks (McGowan et al., 2017). DNA topoisomerases play a critical role in maintaining cellular life by modeling topological changes during DNA replication, transcription, recombination, and chromatin remodeling (Wang, 2002; Yi et al., 2007; Vejpongsa and Yeh 2014b). The DOX-Top2β-DNA complex inhibits DNA replication, stops the cell cycle in G1/G2 and initiates programmed cell death (Tewey et al., 1984; McGowan et al., 2017; Santos and Goldenberg 2018). Animal studies with Top2β knockout (KO) mice have shown that the absence of Top2β reduces DOX-induced cardiotoxicity (Yi et al., 2007; Zhang et al., 2012) in part by reducing mitochondrial dysfunction.

Thus, a wide range of mechanisms for the implementation of DOX cardiotoxic effect is currently being discussed, including oxidative stress, mitochondrial dysfunction, inflammatory processes, ER stress, calcium (Ca2+) dyshomeostasis, apoptosis, ferroptosis, necroptosis, pyroptosis, fibrosis, and autophagy dysregulation (Mitry and Edwards, 2016; Renu et al., 2018). Meanwhile, it can be assumed that all the designated mechanisms are closely involved in the implementation of each other (Minotti et al., 2004).

## Intercalated disc proteins and their role in myocardial function

Cardiac muscle consists of cardiomyocytes interconnected by IDs, which provides synchronous mechanical and electrical activity of individual cardiomyocytes for coordinated excitation and contraction of the heart muscle. In turn, ID includes three types of cellular junctions: gap junctions, desmosomes, and adherens junctions (Muir, 1956; Manring et al., 2018). Desmosomes prevent cardiomyocytes from separating during contraction by anchoring the cell membrane to a network of intermediate filaments, holding cells together, acting like a cell anchor. Adherens junctions (AJs) are actin anchoring sites and connect to the nearest sarcomere to regulate myocardial strength. Gap junctions (GJs) connect the cytoplasm of neighboring cells, allowing action potentials to spread between cardiomyocytes; ions pass between cells, causing depolarization of the heart muscle. Therefore, GJs connect cardiomyocytes metabolically and electrically (Rübsam et al., 2018; Zhao et al., 2019). In this regard, it can be concluded that various disorders, defects of ID proteins or mutations in its gene can be involved in the development of cardiac pathologies such as arrhythmogenic right ventricular cardiomyopathy, dilated cardiomyopathy, hypertrophic cardiomyopathy and restrictive cardiomyopathy, as a result leading to chronic heart failure.

**FIGURE 2:**
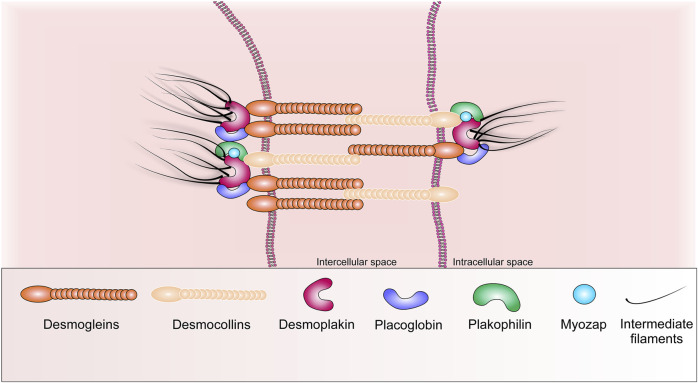
Schematic representation of the basic structural components of desmosome.

The desmosome is based on two transmembrane adhesion molecules belonging to the subfamily of cadherin receptors, these are desmoglein (Dsg) and desmocollin (Dsc) (Manring et al., 2018). Theirs intercellular tails interact with analogues desmosomal cadherin from neighboring cardiomyocytes, while the intracellular tails of Dsg and Dsc bind to desmoplakin (Dsp), myozap protein and armadillo (armadillo family) proteins are placoglobin (Pkg, also called γ-catenin) and plakophilin (Pkp -2) (Figure 2) (Rayns et al., 1969; Bass-Zubek et al., 2009). To date, four isoforms of desmoglein (Dsg 1–4) and three isoforms of desmocollin (Dsc 1–3) have been identified, and their expression exhibits a distinct tissue-specific distribution pattern (Garrod and Chidgey 2008). Thus, desmoglein-2 (Dsg-2) and desmocollin-2 (Dsc-2) are the main isoforms expressed in cardiomyocytes (Garrod and Chidgey 2008). Dsg-2 and Dsc-2 are highly homologous molecules (30 percent amino acid sequence similarity), meanwhile most of the homology found within their extracellular domains. They are also members of the superfamily of Ca2+-dependent molecules and form the core of desmosomal compounds, creating dimers *via* heterophilic interactions (Garrod et al., 2002; Huber, 2003; Dusek et al., 2007).

Pkg interacts with desmosomal cadherins *via* its N-terminal domain, and the arm is repeated near its C-terminus (contains 12 arm repeats). It can also interact with AJ-cadherins, but it has a higher affinity for Dsg-2, which confirms its main localization in desmosomes (Choi et al., 2009). Additionally, Pkg can bind to desmoplakin *via* repeats of its central arm. Pkg, in turn, interacts with desmin-containing filaments. Plakophilin has four splicing products, but Pkp-2 is the most abundant form in human cardiomyocytes. Pkp-2 is able to bind to desmosomal proteins such as desmocollin, desmoplakin, and placoglobin *via* its N-terminal domain, as well as bind to actin and intermediate filament proteins, desmin, and keratin (Al-Amoudi and Frangakis 2008). Furthermore, Pkp-2 interacts with Ankyrin-G, a sodium channel-anchoring protein, and with connexin 43 (Cx43) (Sato et al., 2011), the main GJ component protein. Pkp-2 can interact with PKCα, which is required for phosphorylation and recruitment of desmoplakin into newly formed desmosomes during heart development or repair of cardiac injury (Garrod and Chidgey, 2008).

Dsp is the main protein expressed in the heart. The structure of Dsp is characterized by a central α-helical coiled-coil rod domain, which is surrounded by globular N- and C-termini. Dsp binds the desmin of intermediate filaments and desmosomes. Its N-terminus binds to Pkg and Pkp-2, targeting them to desmosomes. While its C-terminal tail, containing three plakin-repeat domains, provides binding to the desmin protein (Holthöfer et al., 2007). The Myozap protein is also highly expressed in the heart. Myozap colocalizes with β-catenin, N-cadherin and binds directly to Dsp and Pkp-2. Myozap KO in zebrafish leads to cardiomyopathy with severe contractile dysfunction (Seeger et al., 2010). It can be assumed that desmoglein (Dsg), desmocollin, and armadillo family proteins, Dsp-2, and Myozap play an important role in desmosome remodeling and assembly.

**FIGURE 3:**
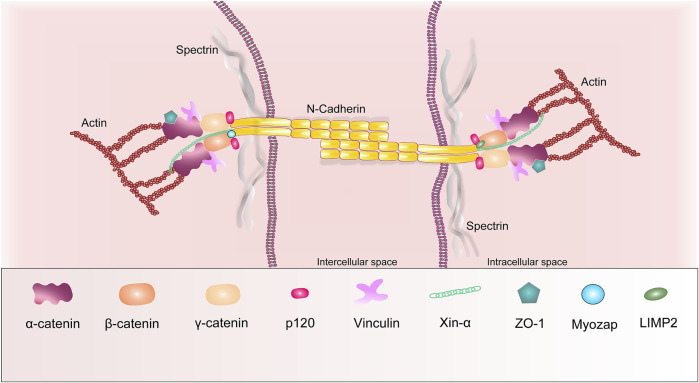
Schematic representation of the basic structural components of the adherens junctions.

AJ, also called fascia adherens, acts as an anchor for myofibrils and contacts the actin filaments of neighboring cardiomyocytes to provide strength and structural support to heart muscle cells. Moreover, AJ regulates mechanical effects on cells by transmitting signals through the actin cytoskeleton. The N-cadherin protein, also known as cadherin-2, plays a major role in AJ (Figure 3). It is a member of the classic cadherin superfamily of transmembrane glycoproteins, including epithelial (E)-, placental (P)-, and neural (N)-cadherin (Bass-Zubek et al., 2009). N-cadherin (N-cad), the only cadherin expressed in the heart, is a transmembrane glycoprotein that mediates calcium-dependent homophilic cell-cell adhesion (Vite and Radice 2014). N-cad forms homodimers with one from neighboring cells in the extracellular space. This provides tissue specificity during development, allowing cells to interact only with cells expressing the same cadherin (Manring et al., 2018; Zhao et al., 2019).

The next important role in AJ is occupied by catenins. They are divided into the armadillo domain-containing family (β-catenin, γ-catenin/placoglobin and p120 catenin) that bind directly to N-cad, and the vinculin homology domain-containing catenin family (αE-catenin and αT-catenin) (Figure 3) (Niessen, 2007; Manring et al., 2018). β-catenin directly binds to the C-terminal cytoplasmic domain of N-cad and binds to actin filaments, interacting with α-catenin and vinculin. The p120 catenin binds the cytoplasmic domain of N-cad. It provides connection of AJ to microtubules by binding to their ends due to two proteins, PLEKHA7 and Nezha (Kaufmann et al., 2000). α-catenins are the key cytoplasmic molecules that bind N-cad to the actin cytoskeleton by interacting with β-catenin, γ-catenin through their N-terminal domains, vinculin or actinin through its C-domain (Janssens et al., 2001). αT-catenin and αE-catenin are the most common isoforms in mammalian cardiac cells of all known α-catenin isoforms (Hirano et al., 1992). Additionally, α-catenin is involved in the interaction with ZO-1. ZO-1 also forms a complex with Cx43 in GJ (Talhouk et al., 2008; Zhao et al., 2019). β-catenin in the heart muscle is part of the N-cad-actin complex and is involved in the Wnt signaling pathway (Nelson and Nusse 2004). Furthermore, β-catenin can be localized to the cytoplasm and nucleus and act as a transcriptional activator. To date, the global role of β-catenin is not clear, since the placoglobin activation compensates loss of β-catenin in the adult heart muscle of various cardiomyocyte-specific β-catenin deficiency models, due to their structural similarity (Zhou et al., 2007).

Nowadays, new proteins of AJ are Xin-α, LIMP2 (lysosomal integral membrane protein 2), spectrin and CAR (Coxsackievirus and adenovirus receptor). Mouse Xin proteins are known to directly bind to N-cad, β-catenin, and actin, thus playing a role in intercellular adhesion and Wnt/β-catenin/N-cadherin-mediated signaling and organization of actin filament assembly (Wang et al., 2011). Wang et al., 2021, wrote that Xin-β functions after angiotensin II signaling and thereby modulates the hypertrophic response in disease (Wang et al., 2011). Spectrin can form tetramers to bind actin filaments at their distal ends (Bennett et al., 2006). The CAR protein is a transmembrane protein that functions as an intercellular adhesion molecule and a common receptor for viruses. CAR interacts with β-catenin and GJ, Cx45, ZO-1 proteins (Bennett et al., 2006; Lim et al., 2008). LIMP2 has been included into AJ proteins due to its contacts with N-cad and its ability to modulate interactions between N-cad and phosphorylated β-catenin in cardiac cells (Schroen et al., 2007).

**FIGURE 4:**
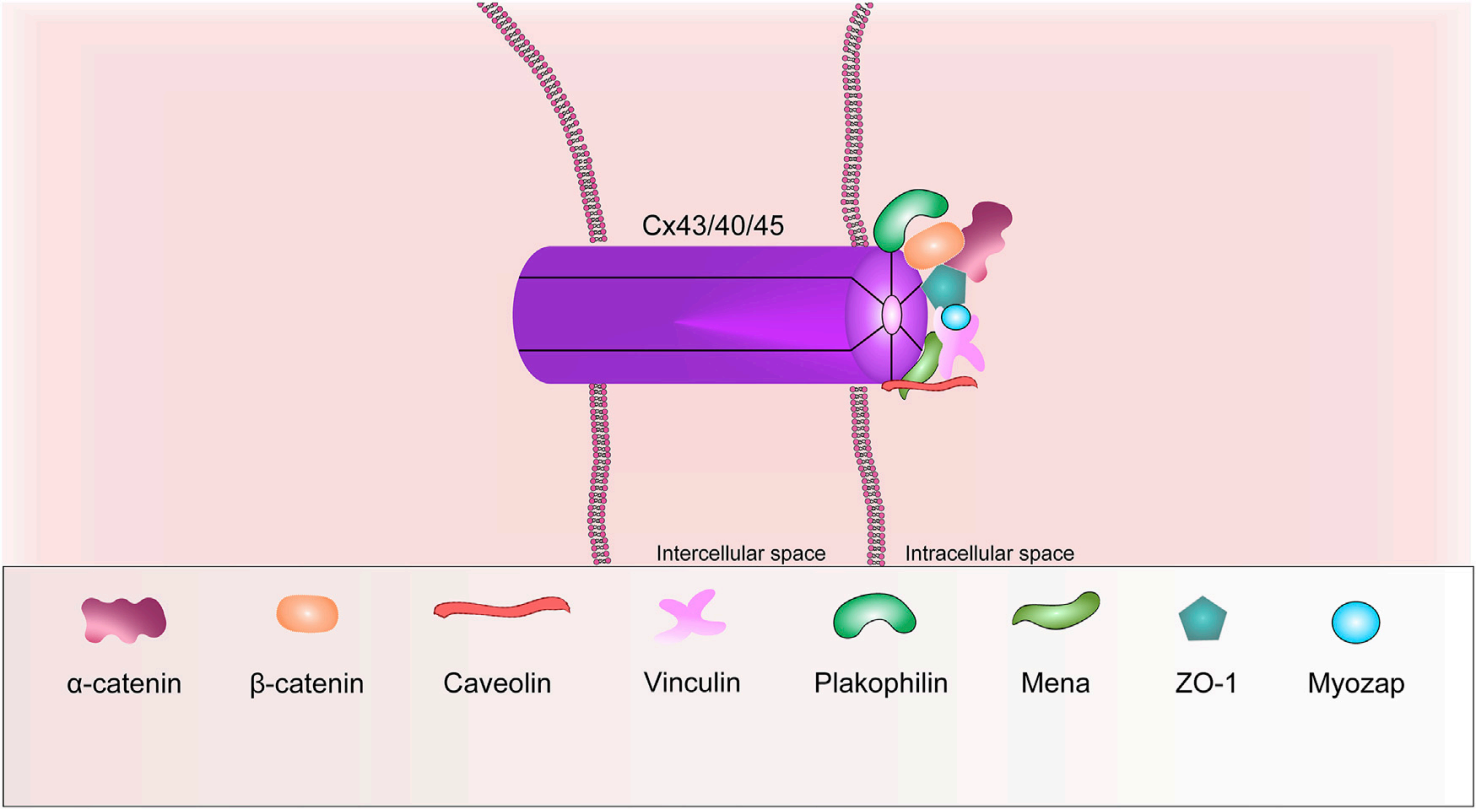
Schematic representation of the basic structural components of the gap junction.

GJ mediates the electrical and metabolic communication of neighboring cardiomyocytes, ensuring their coordinated contraction. The GJ channel in the ventricular cardiomyocyte consists of 12 monomers, six of them form a semi-channel (connexon) in the plasma membrane of each cell. Each connexon is made up of six protein subunits called connexins (Figure 4) (Kumar and Gilula, 1996; Shaw et al., 2007). The superfamily of which consists of at least 21 members. Cx43 is the predominant form expressed in the heart (Severs et al., 2008). In the cardiac muscle, connexins are expressed regionally. Cx43 is found throughout the heart, except for the nodal tissues and parts of the conduction system. Cx43 is the main isoform of connexin in the adult ventricular heart muscle. Cx45 and Cx40 are preferentially expressed in the sinoatrial and atrioventricular nodes, but Cx45 is also co-expressed with Cx43 in the bundle of His and Purkinje fibers. Conversely, Cx43 is predominantly present in the ventricles, but is also co-expressed with Cx40 in the atria heart muscle (Söhl and Willecke 2004).

ZO-1, caveolin, tubulins, mammalian-enabled protein (Mena) are other common GJ proteins. It is known that ZO-1, caveolins and Mena are able to interact with Cx43 *via* their domains. For instance, the ZO-1 protein interacts *via* its PDZ2 domain. Mena interacts with Cx43 *via* its EVH1 domain (Smyth et al., 2010). Ram et al., in 2014 wrote that, the Mena protein can modulate Cx43 remodeling in cardiomyocytes *via* Rac-1 binding (Ram et al., 2014).

It is interesting to note the inextricable relationship of proteins belonging to different groups of cellular junctions. For example, Pkp-2 and Dsg-2 are normally located in desmosomes, also can interact with β-/α-catenin and p120 catenin, which are located in AJs; Pkg is present in both desmosomes and AJ; Pkp2 and Dsc-2 interact with Cx43 in a single complex. Therefore, desmosomes are also inextricably linked to GJs. ZO-1 can connect AJs to GJs by interacting with α-catenin and so on.

Thus, ID is a single functional complex that provides control of cardiac excitability, electrical conductivity, and intercellular adhesion. Dysfunction of any component can lead to the destruction of the entire “house of cards”, leading to the development and progression of heart disease.

## Involvement of intercalated disc proteins in the development of dilated cardiomyopathy

A number of early studies have shown evidence of a direct correlation between the molecular stoichiometry of various the ID proteins and cardiac function. Transgenic mice overexpressing N-cad and E-cad show concomitant upregulation of AJ proteins such as catenin and vinculin and downregulation of Cx-43 in the heart. Moreover, EM images of IDs of these animals show a higher degree of tortuosity of the cardiomyocyte membrane compared to the control (Ferreira-Cornwell et al., 2002). These changes in the composition and architecture of IDs are accompanied by signs of dilated cardiomyopathy (DCM) (Perriard et al., 2003). Analysis of another DCM model showed that desmin KO mice exhibit exactly the same phenotype (Thornell et al., 1997), namely, a higher degree of membrane interdigitation between adjacent cardiomyocytes in IDs in animals compared to wild-type animals. Studies of other changes in DCM models have demonstrated that muscle LIM protein KO (MLP) (Arber et al., 1997) and tropomodulin-overexpressing transgenic (TOT) (Sussman et al., 1998) mice have a more irregular overall shape of cardiomyocytes, increased number of intercellular contacts. Changes in the molecular composition of ID were also found due to increased branching of the cardiomyocytes, while the expression levels of desmosomal proteins did not change, whereas the GJ proteins expression was reduced. Both MLP KO mice and TOT mice show a dramatic upregulation of AJ proteins and therefore myofibrillar attachment (Ehler et al., 2001; Perriard et al., 2003). Thus, there is a strong association between increased the AJ proteins expression in ID and the development of the DCM phenotype.

Desmosomal junction protein genes are the main targets of mutations that cause hereditary heart diseases, not only such as arrhythmogenic right ventricular cardiomyopathy, but the development of the DCM phenotype. These mutations are largely inherited in an autosomal dominant way. They lead not only to a defect of the quantity, integrity, correct localization of desmosomes, but additionally affect the GJ, which ultimately leads to a defect of intercellular conductivity, provoking various kinds of arrhythmias. Numerous national studies *via* next-generation sequencing of 84 genes in 639 patients showed that Pkp-2, myosin-binding protein C-3 and Dsp have the highest number of known mutations. Moreover, Pkp-2, Dsp, Dsc-2 and Dsg-2 are the 4 of the 8 most frequently mutated genes associated with DCM (Haas et al., 2015).

Li and co-authors demonstrated that transgenic mice overexpressing N-cad causes DCM, cardiac calcification, and intracardiac thrombus (Li et al., 2006). These mice showed altered ID structures with increased expression of α-, β-catenin, downregulation of Cx43, and redistribution of vinculin. Furthermore, Kostetskii et al., in 2005 demonstrated that heart-specific N-cad KO causes the absence of identifiable AJ and desmosomes in ID, resulting in to moderate DCM. The authors used the approach of induced N-cad removal from cardiac myocytes after mice have reached maturity. These mice also showed a significant decrease in the expression of catenins (α-, β-, γ-, and p120-) and GJ proteins (Cx40 and Cx43), resulting in slow conduction, spontaneous ventricular arrhythmias, and sudden death (Kostetskii et al., 2005; Li et al., 2006). Thus, reduced expression of the ID proteins may predispose patients to arrhythmias.

Vermij at al. wrote that certain genetic modifications of N-cad, α-catenin, Xin-α or β-catenin in animal models lead to DCM without loss of myocytes and inflammation (Vermij et al., 2017). Other studies using genetically modified mice have shown that deletion or disruption of expression of α-catenin, β-catenin, vinculin genes causes abnormalities in ID function, early DCM, or death (Zemljic-Harpf et al., 2007). Gustafson-Wagner et al. demonstrated that Xinα KO mice had significant postnatal defects in ID ultrastructure, myofilament assembly, and abnormal expression of a number of AJ and desmosome proteins (p120-catenin, β-catenin, N-cadherin, desmoplakin, Cx43) leading to cardiac hypertrophy, cardiomyopathy and conduction disturbances (Gustafson-Wagner et al., 2007). CAR KO mice were characterized by normal development of cardiomyocytes, but subsequently the animals acquired postnatal abnormalities leading to the development of DCM, such as atrioventricular block associated with loss of Cx45, β-catenin, ZO-1 in ID (Lisewski et al., 2008). Mahmoodzadeh et al., in 2006 wrote that β-catenin expression is lost from the ID proteins in end-stage heart failure in humans (Mahmoodzadeh et al., 2006). This suggests that some ID proteins may have unique functions at different stages in the development of cardiac pathologies.

α-E-catenin is widely distributed in the AJ of ID in both epithelial cells and cardiomyocytes. Disruption of its expression or deletion of its gene in mice resulted in progressive DCM and unique right ventricular defects, which included ultrastructural defects in ID and complete loss of vinculin. These animals were also predisposed to rupture of the free ventricular wall after myocardial infarction (Sheikh et al., 2006; van den Borne et al., 2008). At the same time, the expression of other components of AJ, such as β-catenin, γ-catenin and N-cadherin, were not changed in the areas of myocardial damage. Janssens et al., in 2003 reported that the α-T-catenin protein is associated with DCM, but no mutations have been identified so far (Janssens et al., 2003).

Zemljic-Harpf et al. documented that heterozygous normal mice deficient in vinculin were characterized by cardiac dysfunction and increased mortality after acute hemodynamic stress caused by transverse aortic narrowing (Zemljic-Harpf et al., 2007). Authors observed abnormalities in AJ proteins of ID for cardiomyocyte-specific mice with vinculin KO. These abnormalities accompanied by a decrease in the expression of N-cad and β1D integrin, mislocalization of Cx43, which led to sudden death caused by ventricular tachycardia before the age of 3 months. Whereas surviving mice exhibited the DCM phenotype, eventually leading to death at 6 months of age (Zemljic-Harpf et al., 2007). There is also evidence of various mutations and/or deficiencies of vinculin and metavinculin that can carry out the development of DCM. Maeda et al., in 1997 reported that the metavinculin deficiency leading to DCM was caused by a defect in alternative mRNA splicing (Maeda et al., 1997). Olson et al., in 2002 identified three metavinculin mutations (Arg975Trp; Leu954del; Ala934Val) in a study of 350 patients. The authors concluded that the vinculin mutation, Arg975Trp, is found in patients with the phenotype of not only DCM but also obstructive hypertrophic cardiomyopathy (Olson et al., 2002). These studies suggest that interactions between metavinculin and actin may alter the transmission of force within the cardiomyocyte sarcomere and between cardiomyocytes.

Ito at al. in 2021 (Ito et al., 2021) investigated N-cad. They showed that a decrease in the intensity of N-cad immunostaining and scattering of ID are characteristic features of DCM. This fact should help to more accurately diagnose the DCM phenotype and distinguish it from chronic heart failure. Other researchers (Maeda et al., 1997; Olson et al., 2002) report that DCM is associated specifically with a deficiency of metavinculin, an isoform of vinculin found in cardiomyocytes.

Numerous genetic linkage analyzes have revealed the involvement of connexins in at least 14 human diseases, the majority of them can be replicated in mouse models with mutant connexin. Dobrowolski R and Kelly SC showed that Cx43, as the main component of GJ, may be involved in multiple diseases (Dobrowolski and Willecke 2009). Some of these mutations are associated with the development of cardiac disorders, since the levels of Cx43 expression and the amount of GJ are moderately reduced in the heart muscle. However, cardiac conduction is not impaired (Manias et al., 2008). Therefore, a mutation in Cx43 cannot be the only determinant of conduction defects underlying arrhythmogenesis. Mutations in other GJ-associated proteins are still not well understood. However, there is information on genetic disorders, for instance, ZO-1 KO and caveolin KO in mice can cause embryonic lethality and early DCM, respectively (Katsuno et al., 2008). Prevedel et al., in 2017 obtained some interesting data on Cx43 and the impact of HIV infection on it (Prevedel et al., 2017). Authors wrote that HIV infection increases Cx43 expression in the heart. Areas of HIV positive tissue with abnormal Cx43 expression and localization also show calcium overload, sarcofilamental atrophy, and collagen accumulation. This indicates that virus-induced Cx43 activation is involved in damage to the ID proteins, which likely contributes to the high incidence of cardiovascular disease in HIV-infected individuals. Summary information on the involvement of various ID proteins in the development of the DCM phenotype is presented in Table 1.

**TABLE 1:** The role of intercalated disc proteins in the development of the DCM phenotype.

Intercalated disc proteins	Animal model	Effects	Reference
N-cadherin, E-cadherin	Transgenic mice overexpressing N- cadherin and E- cadherin	Upregulation of catenin and vinculin; downregulation of connexin-43; E-cadherin induces nuclear replication and karyokinesis in the absence of cytokinesis, resulting in myocytes with two closely spaced nuclei	Ferreira-Cornwell et al. (2002)
Desmin	Desmin knockout transgenic mice	Degeneration of cardiomyocytes leading to the appearance of areas of accumulation of macrophages, fibrosis and calcification, mainly in the interventricular septum and the free wall of the right ventricle; rupture of the sarcolemma and overcontraction of myofibrils leading to the death of cardiomyocytes.	Thornell et al. (1997)
Actin filaments	Tropomodulin-overexpressing transgenic mice (TOT)	Extensive loss of myofibrillar organization; impaired contractile function	Sussman et al. (1998)
Actin filaments	Muscle LIM protein (MLP) knockout transgenic mice	Defect of the cytoarchitectonics of cardiomyocytes	Arber et al. (1997)
N-cadherin	N-cadherin-overexpressing transgenic mice	Increased α-, β-catenin expression; reduced Cx43 expression; redistribution of vinculin	Li et al. (2006)
N-cadherin	N-cadherin knockout transgenic mice	Disassembly of ID structure (AJ and desmosomes); decreased expression of catenins (α-, β-, γ-, and p120-) and GJ proteins (Cx40 and Cx43), resulting in slow conduction, spontaneous ventricular arrhythmias, and sudden death	Kostetskii et al. (2005)
Vinculin	Vinculin (Vcl) knockout transgenic mice	Abnormal AJ with dissolution of ID structure; reduced expression of the connecting proteins cadherin and beta1D-integrin; incorrect localization of Cx43; sudden death was found in 49% of animals	Zemljic-Harpf et al. (2007)
Xinα	Xinα knockout transgenic mice	ID destruction and disruption of the structure of myofilaments; decreased expression of p120-catenin, β-catenin, N-cad, Cx43, and desmoplakin	Gustafson-Wagner et al. (2007)
LIMP-2	LIMP-2 knockout transgenic mice	Impaired localization of N-cad	Schroen et al. (2007)
CAR	CAR knockout transgenic mice	Defect of electrical conduction between the atrium and the ventricle; decreased expression of Cx45, β-catenin and defect in ZO-1 localization impaired interaction of tight and GJ with altered expression and localization of connexins,	Lisewski et al. (2008)
α-E-catenin	Transgenic mice with α-E-catenin knockout (alpha-E-cat cKO)	Ultrastructural defects of ID and complete loss of vinculin in ID; predisposition of the free wall of the ventricle to rupture after myocardial infarction	Sheikh et al. (2006)
α-E-catenin	Heart samples were collected from autopsies from infarct rupture and control (nonrupture) myocardial infarction patients	Reduced expression and defective localization of α-E-catenin in the region of ID in patients dying from a heart attack	van den Borne et al. (2008)
Vinculin / metavinculin	Metavinculin and vinculin transcripts and protein were analyzed by polymerase chain reaction (PCR) and Western blotting (human heart specimens)	Relationship between metavinculin deficiency and DCM *via* a defect in alternative mRNA splicing	Maeda et al. (1997)
Vinculin / metavinculin	Mutational analyses of the metavinculin-specific exon of vinculin	Vinculin is the gene for DCM	Olson et al. (2002)
N-cadherin	Morphological differences (macroscopic examination, light microscopy, immunohistochemistry, electron microscopy, and gene expression analyses) in the intercalated discs (ICDs) between groups of patients	The disorganized structure of ID contributes to the development of DCM; N-cad immunostaining is useful in determining the pathological diagnosis of DCM.	Ito et al. (2021)
Cx43	Oculo-dento-digital dysplasia (ODDD)	Skin, hair and nail abnormalities	(Debeer et al., 2005; Dobrowolski and Willecke, 2009)
Cx43	Transgenic mouse model of ODDD (Gja1 (Jrt/+)) harbouring a G60S mutation (Cx43 (G60S))	A decrease in the Cx43 protein by 60–80% with a predominant loss of highly phosphorylated forms of Cx43; 50% reduction in conductivity	Manias et al. (2008)
ZO-1, caveolin	ZO-1 knockout (Tjp1 (−/−)) mice	Massive apoptosis in the notochord, the area of the neural tube in embryonic development; angiogenesis defects;	Katsuno et al. (2008)
Cx43	Postmortem human heart tissues obtained from HIV-infected	Overexpression of Cx43; calcium overload, sarcofilamental atrophy and collagen accumulation	Prevedel et al. (2017)
ZO-1	Human cardiac microvascular endothelial cells (HCMECs), altered ERK5 expression using siRNA mediated gene silencing or overexpression of constitutively active MEK5 and ERK5	ERK5 regulates ZO-1 protein	(Wilkinson et al., 2016; Wilkinson et al., 2018)
Claudin-5	Mouse model of muscular dystrophy with cardiomyopathy	Reduced levels of claudin-5, regardless of changes in dystrophin; decreased expression of α-catenin, β-catenin, γ-catenin, desmoplakin, and N-cad	(Mays et al., 2008; Swager et al., 2016)

AJ, adherens junction; ERK5, extracellular signal-regulated kinase 5; GJ, gap junction; ID, intercalated discs.

The listed defects of the ID protein genes can lead to both independently the development of DCM and chronic heart failure, and in combination with other acquired pathologies of the heart muscle. The causes of these pathologies can be both various stresses that initiate cell death, and infections that are involved in a decrease in the level of certain ID proteins and in damage to ID architecture.

There is an important mechanistic link between cell contact protein remodeling and cardiomyopathy. Claudin-5 is present in the side membranes of cardiomyocytes when one is connected to the extracellular matrix, as well as in the endothelial layer of the cardiovascular system. Tessily et al. wrote about a reduction in Claudin-5 in a mouse model of muscular dystrophy that reflect the physiological, histological, and biochemical parameters of heart failure (Mays et al., 2008). Claudin-5 transcript and protein levels are reduced at a very early stage of the disease, and sustained expression of claudin-5 in this model is able to prevent the onset of cardiomyopathy. These data suggest that Claudin-5 may represent a novel treatment target in the early stages of heart failure. Decreased Claudin-5 levels were independent and much more common than changes in other cell junctions and membrane-associated proteins known to be involved in human heart failure, including Cx-43 and dystrophin (Swager et al., 2016). Links between members of the claudin family and matrix metalloproteinases (MMPs) have been identified. It has been shown that MMPs can cleave claudins in tight junctions of endothelial cells (Swager et al., 2016). Claudins are able to activate MMPs, which leads to changes in the adhesion of the extracellular matrix and tumor metastasis in cancer cells. Associations between claudins and MMPs in cancer pathogenesis suggest possible interactions in other disease states that involve extracellular matrix remodeling, including heart failure. The involvement of Claudin-5 in dynamic remodeling suggests that its reduction in 60% of end-stage cardiomyopathy cases may be underestimated. It is possible that claudin-5 undergoes a transient decrease during remodeling but is restored to maintain matrix interactions after remodeling in the most severely affected sites. Thus, it can be assumed that the decrease of claudin-5 level occurs before the onset of damage to the heart, and that the stable expression of claudin-5 in the mouse model prevents the occurrence of cardiomyopathy.

Endothelial cell survival and proliferation are known to be regulated by four known MAPKs: ERK1/2, JNK, P38 and ERK5 (Nithianandarajah-Jones et al., 2012). ERK5 KO in adult mice leads to increased vascular leakage, eventually leading to death within 2–4 weeks. Extracellular signal-regulated kinase 5 (ERK5) regulates tight junction-associated protein ZO-1 (GJ, AJ) and cell permeability. The ERK signaling pathway is involved in T-cell activation, endothelial cell proliferation during angiogenesis, regulation of synaptic plasticity, and phosphorylation of the p53 transcription factor. Anticancer drugs (e.g., anthracyclines) can cause changes in endothelial permeability, which may be the triggering event of drug-induced cardiotoxicity (Wilkinson et al., 2016, Wilkinson et al., 2018). Therefore, DOX can affect the barrier function of endothelial cells in the microvessels of the heart and dermis, leading to the loss of ZO-1 in tight junctions and an increase in cell permeability.

Thus, the ID proteins play a key role in the functioning and remodeling of the heart muscle, which is both under conditions of cardiotoxic effects of drugs and in the development of heart failure.

## Intercalated disc protein signaling pathways

To date, Pkg is the most studied desmosomal protein. It is known that ID carries β-adrenergic receptors and that Pkg is involved in synaptic signaling. In this regard, it has been shown that sympathetic stimulation increases the intercellular cohesion of cardiomyocytes through its second messenger, cAMP (Schinner et al., 2017). Deqiang et al. showed that ventricular arrhythmias are associated with progressive cardiomyopathy. Authors used a mutant mouse model of Pkg (encoded by the JUP gene) (Li et al., 2011). Moreover, transforming growth factor-β (TGFβ) mediated signaling is significantly increased in JUP-mutated cardiomyocytes at an early stage of cardiomyopathy.

Pkp-2 is the only Pkp member expressed in cardiomyocytes. One indirectly regulates transcription by stimulating β-catenin/TCF-mediated transcriptional activity, thereby participating in Wnt-mediated signaling. Garcia Gras et al. reported that suppression of Dsp expression in cardiomyocytes can lead to Pkg nuclear translocation and reduced Wnt/β-catenin signaling through inhibition of TCF/LEF1 transcription factors (Garcia-gras et al., 2006). Pkp-2 can be the main mutational target in the development of various types of cardiac pathologies due to its diverse functional activity (regulation of actin organization, protein synthesis, growth and disease control). This fact is confirmed by Sato et al., who demonstrated in 2011 (Sato et al., 2011) the interaction of Pkp-2 with Cx43 and Ankyrin-G, a cytoskeletal adapter protein that regulates the complex of voltage-gated sodium channels in the heart. Pkp-2 positively regulates EGFR-mediated signaling, thereby reducing cancer cell migration, proliferation, and tumor development (Arimoto et al., 2014). Furthermore, Pkp-2 activates RhoA and PKC-α signaling pathway in keratinocytes (Godsel et al., 2010). The desmosomal protein Myozap may be involved in the activation of serum response factor (SRF) signaling in a Rho-dependent manner in order to bind the ID proteins to the regulation of cardiac genes. This fact and the location of this protein may be related to its possible role in the regulation of severe cardiomyopathy (Seeger et al., 2010). However, its other binding partner, myosin phosphatase-RhoA-interacting protein (MRIP), inhibits this pathway (Seeger et al., 2010). Rangrez et al. showed that myozap can lead to inhibition of MAPK/SRF signaling and activation of β-catenin/GSK-3β and calcineurin/NFAT signaling during pressure overload (Rangrez et al., 2016). Moreover, knockout mice for this protein showed severe cellular and cardiac hypertrophy, cardiac remodeling, increased fibrosis, although the integrity of the heart was not compromised. Thus, it can be assumed that myozap is necessary for proper adaptation to biomechanical stress.

Koichi et al. observed in the intracellular signaling pathway at the synapse (nowadays as the best described model) that N-cadherin-mediated functional disruption of cell contact activated p38 MAPK (Ando et al., 2011). Furthermore, treatment with Aβ(42) reduced cellular expression of N-cad *via* NMDA receptors, which was accompanied by increased phosphorylation of p38 MAPK (Ando et al., 2011). Expression levels of phosphorylated p38 MAPK correlated negatively with the level of N-cad expression in the human brain. Additionally, human brain proteomic analysis revealed a novel interaction between N-cad and JNK-associated leucine zipper protein (JLP), a scaffold protein involved in the p38 MAPK signaling pathway (Ando et al., 2011). α-catenin is a tumor suppressor and can regulate several signaling pathways, for example, inhibit the Wnt/β-catenin pathway, preventing the formation of the β-catenin-TCF-DNA complex and promoting degradation of β-catenin (while p120 catenin has a positive effect on Wnt signal); regulate the Hippo-Yap pathway by blocking YAP dephosphorylation; suppress NF-κB activity by inhibiting ubiquitination and IkB degradation (Li et al., 2015). β-catenin also plays a well-known role in the Wnt signaling pathway as a transcriptional activator (Kim et al., 2013). β-catenin activity is turned on when it is phosphorylated by a specific complex consisting of glycogen synthase kinase-3β (GSK-3β), adenomatous polyposis colon (APC) scaffold proteins, and axin. β-catenin undergoes degradation *via* the ubiquitin-proteasome system, when the Wnt signal is inactive. After activation of the Wnt signal *via* binding to Frizzled (its receptor) leads to accumulation of β-catenin in the nucleus and its interaction with transcription factors of the LEF/TCF family. The LEF/TCF family activates target gene expression and cell growth (Strovel et al., 2000; Nelson and Nusse 2004; Zhao et al., 2019). Differentiated cardiomyocytes do not require a Wnt/β-catenin mediated signal under normal physiological conditions (Bergmann, 2010). Whereas the heart reactivates Wnt/β-catenin signaling and develops cardiohypertrophy under pathological stress. Expression of the Wnt target gene is reduced in β-catenin KO mice, and as a result the animals do not develop cardiac hypertrophy (Park et al., 2005). Thus, β-catenin-mediated signaling plays a critical role in cardiac remodeling in response to stress or injury.

Pkg can work with the same protein complex mentioned above as β-catenin, but additionally Pkg binds to Dsc-2 and Dsg-2, mediating their interaction through Dsp and Pkp-2 with intermediate filaments. Thus, Pkg can compete with β-catenin signaling by inhibiting the expression of Wnt target genes; Pkg can interact with transcription factors and regulate gene expression independently of β-catenin (Aktary et al., 2017).

Cx43, Cx45 and Cx40 are the most abundant of all GJ proteins in cardiomycoites. It is known that phosphorylation at different sites by different kinases can lead to the assembly or disassembly of GJ, which mainly depends on external conditions. For instance, when casein kinase-1 (CK-1) phosphorylates Cx43 at S325/328/330, GJ assembly is activated in a healthy heart (Cooper and Lampe, 2002). Whereas CK-1 is dephosphorylated during ischemia, while protein kinase C (PKC) phosphorylation of S368 is increased (Ek-Vitorin et al., 2006). Four kinases, such as Akt, MAPK, PKC, and Scr, are also thought to play a significant role in the structural regulation of GJ. Cx43 is phosphorylated by Akt, MAPK, PKC, and Scr upon various types of damage or growth factor stimulation, which initiates GJ turnover. Akt kinase is the first to be phosphorylated. ZO-1 protein is a switch to rapidly increase GJ communication, potentially leading to the initiation of an ischemic injury response in the heart (Solan and Lampe 2016). Scr activation also leads to phosphorylation of Cx43 and initiation of GJ assembly; in addition, Scr can interact and compete for one binding site with ZO-1. PKC in turn leads to inhibition of GJ assembly by phosphorylation of Cx43 (S368), resulting in GJ channel closure (Kieken et al., 2009). There is some interesting information regarding the relatively new CAR protein, a receptor for two cardiotropic viruses. CAR interacts with Cx45, ZO-1, and β-catenin. Its overexpression in cardiomyocytes causes increased GSK-3β phosphorylation and Akt activation, leading to cardiomyopathy, *via* phosphorylation of the p44/p42 MAPK and JNK pathways (Caruso et al., 2010).

Thus, the molecular mechanisms of interaction between ID components and the influence of their signaling pathways on the cell and cellular relationship in the tissue play a decisive role in the formation of cardiotoxicity. The search of ways to regulate molecular signaling pathways for myocardial ID proteins can become the basis for creating new therapeutic approaches to the treatment and prevention of cardiac pathologies.

## Effect of doxorubicin on intercalated disc proteins

Due to the fact that GJ channels are responsible for direct intercellular communication and remodeling of the organization of the GJ, defects in their structure can manifest in the form of heart disease. Zhang et al. demonstrated for the first time in 2011 that DOX suppresses cardiac expression of Cx43 and Cx45, leading to loss of Cx43/Cx45 junction channels and subsequent spatial GJ remodeling, resulting in progressive cardiac dysfunction (the development of DOX-induced cardiomyopathy) and left ventricular remodeling (Zhang et al., 2011). The authors also found that DOX induces activation of the JNK pathway in the heart. Thus, the DOX-induced downregulation of Cx43 and Cx45 may be mediated by the JNK pathway. Treatment with S-diclofenac or NaHS reduced JNK phosphorylation in the heart of DOX-treated mice. Moreover, a number of authors demonstrated the presence of Cx43 in cardiac mitochondria (Pecoraro et al., 2015, 2018; Elhadidy et al., 2020), which may also play an additional role in protecting cardiac cells from DOX-induced toxicity by overexpressing Cx43, causing a decrease in the production of mitochondrial ROS (Pecoraro et al., 2020). Pecoraro et al. showed that DOX causes a defect of Ca2+ homeostasis, which manifests after a single injection, and affects the expression and localization of Cx43 (Elhadidy et al., 2020). The authors also wrote that increased phosphorylation of Cx43 and Cx43 leads to a decrease in mitochondrial Ca2+ accumulation (Elhadidy et al., 2020).

Wilkinson et al. showed that ERK5 regulates the formation of tight junctions, through interaction with the ZO-1 adapter protein, and controls the permeability of endothelial cells of cardiac microvessels (Wilkinson et al., 2018). ERK5 activation mediated by statins leads to translocation of ERK5 to the plasma membrane, regulation of tight junction formation, and decreased endothelial cell permeability induced by DOX treatment (Wilkinson et al., 2016; Luu et al., 2018). How DOX reduces ZO-1 expression in the absence of endothelial cell apoptosis remains to be elucidated.

Thus, there are just a few works presented in the field concerned to the study of DOX-induced cardiotoxicity in terms of structural, functional, and molecular signaling disorders of myocardial ID proteins. Despite this, the involvement of ID proteins in the development of DOX-induced cardiotoxicity has been shown in the literature.

## Cardioprotective role of SIRT1

Sirtuins are NAD + -dependent deacylases capable of responding to DNA damage, oxidative stress, and inflammation. Sirtuin activation triggers nuclear transcription programs that increase metabolic efficiency, as well as enhance mitochondrial oxidative metabolism and resistance to oxidative stress, cellular survival (Imai and Guarente, 2014). In the 90s, their ability to increase the lifespan of yeast (silent information regulator, SIR2) was first discovered. In this regard, it has been shown that activation of Sir2 expression reduces DNA damage and increases the lifespan of yeast (Kaeberlein et al., 1999). Afterwards, sirtuins began to be attributed to the third class of histone deacetylases, requiring NAD + as a cofactor for the reaction to occur (Du et al., 2011). Sirt1-deficient mice show marked cardiac developmental defects and do not survive the postnatal period (Cheng et al., 2003). These molecules are found in all organisms, from bacteria to eukaryotes, and their sequences are quite conservative. Seven SIRT proteins are known to be encoded in the human genome (Bugger et al., 2016; Kane and Sinclair, 2018). SIRT1,2,6,7 of mammals are located in the nucleus, SIRT1,2–in the cytoplasm, SIRT3,4,5–in mitochondria, where they deacetylate non-histone proteins in the process of regulation of various metabolic processes (Cantó et al., 2013; Covarrubias et al., 2021). SIRT1 is the most well-studied sirtuin. SIRT1 also modulates transcription factors such as p53, clear factor kappa-light-chain-enhancer of activated B cells (NF-kB) (Yeung et al., 2004), FOXOs, and peroxisome proliferator-activated receptor gamma coactivator 1-alpha (PGC1α) (Rodgers et al., 2005; Mouchiroud et al., 2013). Moreover, SIRT1 regulates DNA repair proteins such as Poly ADP-ribose polymerase 1 (PARP1) (Rajamohan et al., 2009). In summary, the sirtuin deacylation reaction involves the removal of the acetyl group from target substrates by converting NAD + to nicotinamide (NAM) and O-acetyl-ADP-ribose (Covarrubias et al., 2021). The deacetylase activity of Sirt1 is regulated by the presence of NAD+.

Over the past decade, knowledge of the functional activity of NAD + has expanded. It has been shown the role of NAD + as a key modulator of cellular signaling and survival pathways in the body, in addition to NAD + can catalyze electron transfer in redox reactions. The discovery of two very interesting facts, namely: 1) NAD+ is an important substrate of sirtuin metabolism (Kane and Sinclair, 2018; Rajman et al., 2018); 2) description of the contribution of nicotinamide riboside (NR) and its kinases (NRKs) to NAD + metabolism (Bieganowski and Brenner, 2004; Yoshino et al., 2018) has revived interest in NAD + as a potential modulator of longevity and health. It has been shown that NAD + reserves in cells are replenished due to the work of several metabolic pathways: *de novo* synthesis from dietary tryptophan; through the disposal pathways of NAD + precursors such as nicotinamide (NAM), NR and nicotinic acid (NA) (*the Preiss-Handler, Salvage, Core Recycling Pathways*) (Croft et al., 2020; Podyacheva and Toropova 2021). Thus, NAD + levels can be increased by appropriate precursors (NR, NAM, NMN); by increasing levels of enzymes involved in NAD + synthesis pathways such as nicotinamide phosphoribosyltransferase (NAMPT) (Yang et al., 2007) or inhibit NAD + consuming enzymes, including the CD38/157 differentiation cluster, TIR motif-containing protein 1 (SARM1), and poly-ADP-ribose polymerases (PARPs) (Chini, 2009; Bai and Cantó, 2012; Brown et al., 2016). An increase in NAD + levels is highly correlated with sirtuin activation (Masri et al., 2014).

The functional activity of SIRT1 covers a large area of influence: regulation of oxidative stress, inflammatory response (Brookins Danz et al., 2009; Xu et al., 2020), role in mitochondrial function, apoptosis regulation (Cappetta et al., 2016; Yuan et al., 2018; Chen et al., 2020), endoplasmic reticulum stress, (Lou et al., 2015), role in the fibrosis formation (Cappetta et al., 2016) and so on. All of these functions are mediated by certain signaling pathways (SIRT1/AMPK, SIRT1/p53, SIRT1/NF-κB, SIRT1/p38 MAPK, SIRT1/FOXOs, SIRT1/TGF-β, SIRT1/PGC-1α), which play an important role in cardiac pathologies, including DOX-induced cardiotoxicity development. Let us pay special attention to the signaling pathways, which are also in contact with the known molecular regulation of the ID proteins of the cardiac muscle.

NF-κB is the most well-known regulator of inflammatory and immunological responses, which plays a key role in the development of DOX-induced cardiotoxicity. Recent studies have shown that DOX can upregulate NF-κB expression, causing inflammation in the heart (Bin Jardan et al., 2020). Kauppinen et al. showed that SIRT1 inhibits NF-κB expression through deacetylation of the RelA/p65 NF-κB subunit on Lys310 to regulate inflammatory phases and energize metabolic (Kauppinen et al., 2013). Han et al., in 2020 wrote that the SIRT1/NF-κB pathway is also involved in the inhibition of myocardial apoptosis to protect H9c2 cells from hypoxia-induced damage (Han et al., 2020). In a 2018 study, Yuan et al. found that overexpression of SIRT1 may have an anti-inflammatory effect to improve DOX-induced cardiotoxicity by reducing TNF-α levels and NF-kB nuclear translocation (Yuan et al., 2018). Bagul et al. determined that SIRT1 activation by resveratrol pretreatment could inhibit NF-κB expression, reducing oxidative stress and cardiac inflammation in diabetic rats (Bagul et al., 2015).

p38 MAPK activity is mediated by upstream kinases known as MAPKkinases (MKK), such as MKK3 and MKK6. p38 MAPK is activated by inflammatory response and osmotic (Martin et al., 2015). Studies by authors such as Kojonazarov et al., 2017, Wang et al., 2012, Becatti et al., 2012 and Ruan et al., 2015 (Becatti et al., 2012; Wang et al., 2012; Matsushima and Sadoshima, 2015; Kojonazarov et al., 2017) demonstrated that p38 MAPK activation was involved in several pathological heart changes, and p38 MAPK inhibition could improve cardiac fibrosis, right ventricular hypertrophy with pressure loading, and oxidative stress of the heart. It has also been reported that inhibition of the p38 MAPK/NF-κB pathway can reduce levels of inflammatory factors: IL-1β, IL-6, IL-17, and TNF-α, which in turn protects H9c2 cells from DOX toxicity (Guo et al., 2013). Overexpression of SIRT1 can attenuate mitochondrial dysfunction, oxidative stress, and apoptosis, improving cardiomyocyte health. SIRT1 acts as a negative regulator of p38 MAPK, participating in the treatment of DCM, including DOX-induced cardiotoxicity, by reducing p38 MAPK and JNK phosphorylation, as well as increasing ERK phosphorylation (Becatti et al., 2012).

TGF-β is a multipotent cytokine. It takes part in modulating cell growth, proliferation, differentiation and apoptosis. Moreover, TGF-β regulates the production of extracellular matrix, including collagen and fibronectin (Ignotz and Massague, 1986). SIRT1 activation by tetrahydrocurcumin may inhibit the TGF-β1/SMAD3 pathway to exert antifibrotic function, abolish TGF-β-induced collagen synthesis, and improve DOX-induced cardiotoxicity (Schiller et al., 2004; Hu et al., 2018). In turn, TGF-β stimulates downstream SMAD targets, causing overexpression of profibrotic genes. Resveratrol activation of SIRT1 reduced TGF-β levels and the pSMAD3/SMAD3 ratio, as well as decreased fibronectin and type I collagen expression. SIRT1 subsequently inhibited cardiac fibrosis and fibroblast activation, improving diastolic dysfunction and heart failure in a rat model of DOX-induced cardiotoxicity (Cappetta et al., 2016). Thus, antifibrotic function mediated by SIRT1/TGF-β may play an indispensable role in the treatment of DOX-induced cardiotoxicity.

AMPK activation increases ATP-producing catabolism, including glucose metabolism and fatty acid oxidation, and reduces ATP-consuming anabolism, such as protein and lipid synthesis, to maintain cellular energy stores (Herzig and Shaw 2018). Moreover, AMPK activity leads to advanced mitochondrial biogenesis by increasing PGC-1α expression (Zong et al., 2002). Gratia et al. wrote that DOX can inhibit AMPK phosphorylation and activate downstream mTOR, causing an abnormal cardiac phenotype (Gratia et al., 2012). One study showed that SIRT1/AMPK can improve cardiac function, reduce the size of myocardial infarction through deacetylation of liver kinase B1 (LKB1) and subsequent AMPK activation (Wang et al., 2018). Wang et al. demonstrated that fibroblast growth factor 21 (FGF21) can promote interaction of SIRT1 with LKB1 to enhance LKB1 deacetylation and subsequent AMPK activation to prevent DOX-induced oxidative stress, inflammation and apoptosis, as well as improve cardiac dysfunction by increasing nuclear expression of NRF2 and inhibition of NF-κB both *in vivo* and *in vitro* (Wang et al., 2017; Wang(a) et al., 2021). The AMPK/LKB1 activation pathway regulates lipid, glucose, and cholesterol metabolism in the liver, muscle, and adipose tissue. Additionally, SIRT1/ LKB1 signaling pathway can improve the metabolic remodeling of ventricular cardiomyocytes of the heart induced by aldosterone stimulation. AMPK is also able to activate SIRT1, modulating intracellular NAD + metabolism (Liu et al., 2019; Dziewulska et al., 2020). Huang et al. described an AMPK-SIRT1-TFEB pathway leads to improved regulation of autophagy by stimulating lysosomal biogenesis (Huang et al., 2019). The SIRT1/PPARα signaling pathway regulates the number of genes expression responsible for lipid and lipoprotein metabolism in tissues with a high level of fatty acid catabolism, such as the liver, heart, and muscle (Li et al., 2011). Upadhyay et al. demonstrated that DOX, by blocking SIRT1, suppresses the expression of PPAR-α and PPAR-γ in cells, leading to disruption of lipid homeostasis and cardiac metabolism (Upadhyay et al., 2020).

Thus, SIRT1 and NAD + play an important role in maintaining the homeostasis of metabolic processes and the function of the cardiac system. The use of SIRT-NAD + as a therapeutic target may lead to the creation of radically new approaches to the prevention and treatment of cardiac pathologies.

## Effect of SIRT1 on intercalated disc proteins as a mechanism of cardioprotection under doxorubicin exposure

A healthy heart is able to compensate for various changes associated with volume or pressure overload *via* dilatation and hypertrophy under normal physiological conditions. Whereas with the development of DCM, DOX-induced cardiotoxicity, and chronic heart failure, the heart muscle undergoes irreversible remodeling (Bray et al., 2018; Markham et al., 2020). While remodeling of the left ventricular wall is observed, which becomes thinner with time (dilatation develops). As a result, this process leads to a defect of myocardial contractility with the development of cardiac dysfunction, which is accompanied by arrhythmia, thromboembolism and can lead to death (Pfeffer and Braunwald, 1990; Menna et al., 2012; Matsushima and Sadoshima 2015). Cytoskeletal abnormalities may be a common feature of this disease since DOX-induced cardiomyopathy is associated with impaired contractility and changes in the shape of cardiomyocytes.

The involvement of known signaling pathways such as integrin−/−catenin-mediated signaling, which is a key element of the Wnt signaling pathway, may play an important role (Ehler et al., 2001). Elevated levels of intracellular free Ca2+ may be another possible factor in some types of cardiomyopathies, including DOX-induced cardiomyopathy (Minamisawa et al., 1999). Since Ca2+ is involved in many cellular processes, it is difficult to determine which pathway is affected by excess free Ca2+ in the cytoplasm of cardiomyocytes in DCM. It can be assumed that changes occurring at the level of the cardiomyocytes, namely, in the structure and colocalization of the ID protein composition, can also contribute to the heart dysfunction. Processes characteristic of DCM phenotype, such as increased expression of AJ proteins, increased membrane coagulation and interdigitation between neighboring cardiomyocytes, lead to a decrease in the flexibility of the contractile tissue and an increase in its rigidity. Changes in the localization or expression levels of Cx43 can further impair the contractility of the heart tissue, preventing the correct synchronization required for controlled contraction. Such defects can lead to arrhythmias typical of heart failure. Thus, changes in the molecular composition of ID can be directly correlated with physiological changes.

**FIGURE 5:**
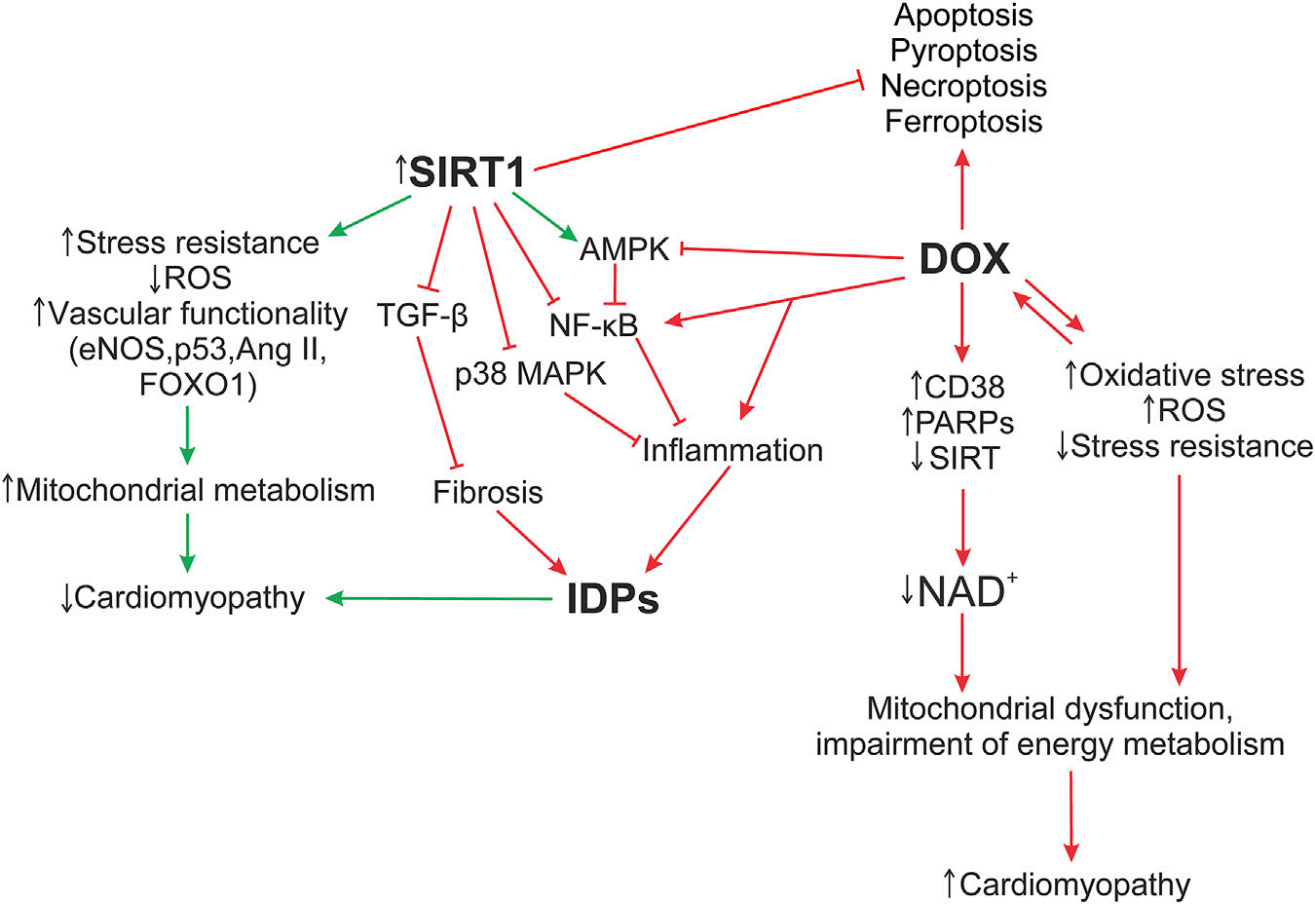
Hypothesis of the SIRT1 protective effect on myocardial ID proteins in DOX-induced cardiotoxicity. The formation of DOX-induced cardiotoxicity occurs due to the triggering of oxidative stress, inflammatory processes *via* the activation of TLR4/MAPKs/NF-κB pathways, and doxorubicin also inhibits the work of topoisomerase 2β. As a result, DNA damage increases, the work of cell compartments (mitochondria, sacroplasmic reticulum) is disrupted, and the structure of the myocardial ID is destroyed. Additionally, activation of DNA repair enzymes, PARPs, CD38, leads to a decrease in the functional activity of sirtuins, disruption of the work of Electron transport chain Complex I, which uses NADH as an electron donor. Damage of Complex 1 and ATP production leads to the accumulation of ROS, which increase the level of oxidative stress. Influencing SIRT1 initiates systems that reduce DOX-induced cardiotoxicity, such as antioxidant defense systems that restore mitochondrial biogenesis damaged by oxidative stress; autophagy activation systems that are disrupted by DOX, leading to accumulation of non-degradable autolysosomes; activates SIRT1/AMPK and inhibits SIRT1 p38 MAPK/NF-κB/TGF-β signaling pathways that contribute to the normalization of the regulation of inflammatory responses, improve the functioning of ID structures, modulate apoptotic processes, and exert antifibrotic regulation. It is interesting to note the possible role of SIRT1 in the regulation of switching between the processes of necroptosis, pyroptosis, and apoptosis. However, these mechanisms are not considered in this study. AMPK, 5′ AMP-activated protein kinase; Ang II, angiotensin II; CD38, cADP-ribosesynthases; DOX, doxorubicin; eNOS, Endothelial NOS; Forkhead box protein O1; IDPs, intercalated disc proteins; NF-κB, nuclear factor kappa-light-chain-enhancer of activated B cells; p38 MAPK, p38 mitogen-activated protein kinases; p53, tumor protein P53; PARPs, poly-ADP-ribose polymerases; ROS, reactive oxygen species; SIRT, silent information regulator 2 (Sir2); TGF-β, transforming growth factor beta; FOXO1.

The functioning of SIRT1, as well as the ID proteins, is mediated by signaling pathways. A defect in one ID protein leads not only to a local change in ID architecture, but also further provokes a damage of other components of the myocardial structure, carrying out the development of DCM phenotype. Signaling pathways such as SIRT1/NF-κB, SIRT1/p38 MAPK, SIRT1/TGF-β, SIRT1/AMPK may play an important role in maintaining the functioning of cardiomyocytes, influencing the functioning of ID structures during the cardiomyopathy development. Figure 5 shows the hypothesis of the SIRT1 protective effect on myocardial ID proteins in DOX-induced cardiotoxicity.

DOX enhances the pro-inflammatory factor NF-κB expression, (Bin Jardan et al., 2020), while SIRT1 inhibits it, normalizing the regulation of inflammatory responses and energy supply of cellular metabolic processes (Kauppinen et al., 2013). The AJ protein, α-catenin, is additionally able to downregulate NF-κB activity, reducing tumor exposure and positively affecting the Wnt signal and the Hippo-Yap pathway, which controls the regulation of cell proliferation and apoptosis (Li et al., 2015). Activation of the SIRT1/AMPK pathway can also induce NF-κB inhibition, preventing DOX-induced oxidative stress, inflammation, apoptosis, and additionally, modulate intracellular NAD + metabolism (Zong et al., 2002; Herzig and Shaw 2018). In case of myocardial cadherin defect, N-cad, the p38 MAPK signaling pathway is activated, which can lead to pathological changes in the heart (Ando et al., 2011). Whereas overexpression of SIRT1 works as a negative regulator of p38 MAPK, attenuating mitochondrial dysfunction and oxidative stress by reducing p38 MAPK and JNK phosphorylation. Activation of the p38 MAPK pathway may also affect the function of GJ proteins. Various types of damage lead to phosphorylation of Cx43, ZO-1 by MAPK kinases, which induces GJ turnover and myocardial remodeling (Caruso et al., 2010; Solan and Lampe 2016). In general, the ability of SIRT1 to inhibit p38 MAPK/NF-κB pathways reduces the levels of other inflammatory cytokines in DOX-induced cardiomyopathy, such as IL-1β, IL-6, IL-17, and TNF-α (Guo et al., 2013). Mutation in the desmosome protein, Pkg, increases TGFβ-mediated signaling in the early stages of cardiomyopathy (Li et al., 2011), leading to increased expression of fibronectin and type I collagen. While SIRT1 activation reduces TGF-β levels, exerting antifibrotic regulation (Schiller et al., 2004; Cappetta et al., 2016; Hu et al., 2018). It is important to note that ID is a single structural and functional complex of interconnected proteins. The triggering of a pathological signal from one defective protein can lead to the initiation of damage to the others. SIRT1 activation can contribute to the normalization of not only metabolic dysregulation and myocardial inflammation processes inside the cell, but also have a positive effect on the cytoskeletal structures of the cell itself, without them electrical conductivity, cardiac muscle excitability, and intercellular adhesion will be impaired.

Thus, SIRT1 can be considered as a pharmacological target, the activation of one can help reduce doxorubicin-induced cardiotoxicity. It can be assumed that the mechanisms of influence of SIRT1 on intercalated disc proteins are involved in the implementation of the cardioprotective effect.

## Glossary

**AJ**: adherens junctions
**AMPK**: 5′ AMP-activated protein kinase
**Ang II**: angiotensin II.
**ATF6/4**: activating transcription factor 6/4
**CD38**: cyclic ADP-ribose synthase
**CHOP**: C/EBP homologous protein
DCM: dilated cardiomyopathy
**DOX**: doxorubicin
**DOXO**: semiquinone form of doxorubicin
**eNOS**: endothelial NOS
**ETC**: electron transport chain
**FOXO1**: forkhead box protein O1
**GJ**: gap junction
**GRP78**: 78-kDa glucose-regulated protein precursor
**ID**: intercalated disc
**IL-6/17/1β**: interleukin 6/17/1β
**IRE-BP**: iron-responsive element-binding proteins
**KO**: knockout
**MAPKs**: mitogen-activated protein kinases
**mPTP**: mitochondrial permeability transition pore
**NA**: nicotinic acid
**NAD+**: nicotinamide adenine dinucleotide, oxidized form
**NAM**: nicotinamide
**NAMPT**: nicotinamide phosphoribosyltransferase
**NF-κB**: nuclear factor kappa-light-chain-enhancer of activated B cells
**NMN**: nicotinamide mononucleotide
**NR**: nicotinamide riboside
**NRK**: nicotinamide riboside kinase
**p38 MAPK**: p38 mitogen-activated protein kinases
**p53**: tumor protein P53
**PARP**: poly(ADP-ribose) polymerase
**PERK**: (PKR)-like endoplasmic reticulum kinase
**SIRT**: sirtuin deacetylase (Silent Information Regulator 2 (Sir2))
**TGF-β**: transforming growth factor beta
**TLR4**: toll-like receptor 4
**TNF-α**: tumor necrosis factor
