# Erratum: SIRT1 activation and its effect on intercalated disc proteins as a way to reduce doxorubicin cardiotoxicity

**DOI:** 10.3389/fphar.2023.1154384

**Published:** 2023-02-09

**Authors:** Frontiers Production Office

**Affiliations:** Frontiers Media SA, Lausanne, Switzerland

**Keywords:** adherens junctions, desmosomes, doxorubicin cardiomyopathy, gap junction, heart failure, intercalated discs, NAD+, sirtuins

Due to a production error, the reference for “Ito et al., 2021” was incorrectly written as “Ito, Y., Yoshida, M.,Masuda, H.,Maeda, D., Kudo-Asabe, Y., Umakoshi,M., et al. (2021). Neovasculature can be induced by patching an arterial graft into a vein: A novel *in vivo* model of spontaneous arteriovenous fistula formation. Sci. Rep. 11, 3156–3213. doi:10.1038/s41598-018-21535-2”. It should be “Ito, Y., Yoshida, M., Masuda, H., Maeda, D., Kudo-Asabe, Y., Umakoshi, M., et al. (2021). Disorganization of intercalated discs in dilated cardiomyopathy. *Sci. Rep.* 11, 1–13. doi: 10.1038/s41598-021-90502-1”.

The publisher apologizes for this mistake. The original version of this article has been updated.

